# Teledentistry from research to practice: a tale of nineteen countries

**DOI:** 10.3389/froh.2023.1188557

**Published:** 2023-06-16

**Authors:** Maha El Tantawi, Walter Yu Hang Lam, Nicolas Giraudeau, Jorma I. Virtanen, Cleopatra Matanhire, Timothy Chifamba, Wael Sabbah, Noha Gomaa, Sadeq Ali Al-Maweri, Sergio E. Uribe, Simin Z. Mohebbi, Noren Hasmun, Guangzhao Guan, Ajith Polonowita, Sadika Begum Khan, Massimo Pisano, Passent Ellakany, Marwa Mohamed Baraka, Abdalmawla Alhussin Ali, José Eduardo Orellana Centeno, Verica Pavlic, Morenike Oluwatoyin Folayan

**Affiliations:** ^1^ Department of Pediatric Dentistry and Dental Public Health, Faculty of Dentistry, Alexandria University, Alexandria, Egypt; ^2^ Prosthodontics, Restorative Dental Sciences, Faculty of Dentistry, The University of Hong Kong, Hong Kong, Hong Kong SAR, China; ^3^ CEPEL, CNRS, University of Montpellier, Montpellier, France; ^4^ Institute of Dentistry, Faculty of Medicine, University of Turku, Turku, Finland; ^5^ Department of Oral Health, Faculty of Medicine and Health Sciences, University of Zimbabwe, Harare, Zimbabwe; ^6^ Centre for Host Microbiome Interactions, King’s College London, London, United Kingdom; ^7^ Schulich School of Medicine & Dentistry, Western University, London, ON, Canada; ^8^ Department of Pre-Clinical Oral Health Sciences, College of Dental Medicine, QU Health, Qatar University, Doha, Qatar; ^9^ Department of Conservative Dentistry and Oral Health, Riga Stradins University, Riga, Latvia; ^10^ School of Dentistry, Universidad Austral de Chile, Valdivia, Chile; ^11^ Baltic Biomaterials Centre of Excellence, Headquarters at Riga Technical University, Riga, Latvia; ^12^ Research Center for Caries Prevention, Dentistry Research Institute, and Department of Community Oral Health, School of Dentistry, Tehran University of Medical Sciences, Tehran, Iran; ^13^ Department of Oral Sciences, Faculty of Dentistry, University of Otago, Dunedin, New Zealand; ^14^ Department of Prosthetic Dentistry, Faculty of Dentistry, University of the Western Cape, Cape Town, South Africa; ^15^ Department of Medicine, Surgery and Dentistry “Scuola Medica Salernitana”, University of Salerno, Baronissi, Italy; ^16^ Department of Substitutive Dental Sciences, College of Dentistry, Imam Abdulrahman Bin Faisal University, Dammam, Saudi Arabia; ^17^ Orthodontics Department, Dental Faculty, Sirte University, Sirte, Libya; ^18^ Faculty of Dentistry, Public Health Research Institute, University of Sierra Sur, Oaxaca, México; ^19^ Department of Periodontology and Oral Medicine, Faculty of Medicine, University of Banja Luka, Banja Luka, Bosnia and Herzegovina; ^20^ Department of Child Dental Health, Obafemi Awolowo University, Ile-Ife, Nigeria

**Keywords:** teledentistry, global oral health, oral health policies, COVID-19, healthcare system

## Abstract

**Aim:**

The COVID-19 pandemic has accelerated teledentistry research with great interest reflected in the increasing number of publications. In many countries, teledentistry programs were established although not much is known about the extent of incorporating teledentistry into practice and healthcare systems. This study aimed to report on policies and strategies related to teledentistry practice as well as barriers and facilitators for this implementation in 19 countries.

**Methods:**

Data were presented per country about information and communication technology (ICT) infrastructure, income level, policies for health information system (HIS), eHealth and telemedicine. Researchers were selected based on their previous publications in teledentistry and were invited to report on the situation in their respective countries including Bosnia and Herzegovina, Canada, Chile, China, Egypt, Finland, France, Hong Kong SAR, Iran, Italy, Libya, Mexico, New Zealand, Nigeria, Qatar, Saudi Arabia, South Africa, United Kingdom, Zimbabwe.

**Results:**

Ten (52.6%) countries were high income, 11 (57.9%) had eHealth policies, 7 (36.8%) had HIS policies and 5 (26.3%) had telehealth policies. Six (31.6%) countries had policies or strategies for teledentistry and no teledentistry programs were reported in two countries. Teledentistry programs were incorporated into the healthcare systems at national (*n* = 5), intermediate (provincial) (*n* = 4) and local (*n* = 8) levels. These programs were established in three countries, piloted in 5 countries and informal in 9 countries.

**Conclusion:**

Despite the growth in teledentistry research during the COVID-19 pandemic, the use of teledentistry in daily clinical practice is still limited in most countries. Few countries have instituted teledentistry programs at national level. Laws, funding schemes and training are needed to support the incorporation of teledentistry into healthcare systems to institutionalize the practice of teledentistry. Mapping teledentistry practices in other countries and extending services to under-covered populations increases the benefit of teledentistry.

## 1. Introduction

Teledentistry is the use of information and communication technology (ICT) for dental consultations, diagnosis and treatment planning, including the transmission of clinical information and images between an oral health professional and patient or between two health professionals, including at least one oral health professional, who are separated by distance (1). There are some related terms, including digital health which is the use of digital technologies to improve health (2, 3). Digital health initially focused on improving existing communication processes and later, extended to new mobile health applications when powerful mobile devices appeared. Also, electronic health is the use of ICT to support health and health-related fields, including healthcare services, health surveillance, health literature and health education, knowledge and research (4), and empowering patients (5, 6).

The first mention of the term teledentistry in a MEDLINE indexed paper was in an American study comparing the resolution of digitized radiographs of the temporomandibular joint in 1996 (7). Since then, about seven papers have been published annually till 2019 (8). The COVID-19 pandemic accelerated the growth in the number of publications on teledentistry with about an average of 86 papers indexed in MEDLINE annually between 2020 and 2022. It is estimated that 61% of all teledentistry papers were published between 2020 and 2022 (8).

The growth in teledentistry research indicates an interest in generating evidence to guide the use of teledentistry in clinical practice. Teledentistry supports access to dental care, especially in remote areas, in places with dentists' shortage and for difficult to reach groups (1). It is useful for consultation between patient and dentist, between general dental practitioners and specialists, for patient referral (9) and for reducing the need for in-person dental visits with cost and time saving for dentists and patients (10). Challenges facing the widespread implementation of teledentistry include the need for a robust technology infrastructure, training, supportive re-imbursement systems and policies to protect patient confidentiality and guide practice (10). The COVID-19 pandemic may have paved the way for integrating teledentistry into national healthcare systems although between-countries variation in technological infrastructure, healthcare systems characteristics and regulatory bodies might limit the speed of the integration.

National policies and guidelines for teledentistry foster the development of an environment that facilitates the practice of teledentistry. Despite the growing number of studies describing teledentistry projects, and attitudes toward teledentistry and its benefits (11), there is little evidence about the progress made to institutionalize the practice of teledentistry in-country. Sharing knowledge about national policies and processes governing the incorporation of teledentistry into healthcare systems in different countries and different context can further strengthen the global process of developing frameworks for the practice of teledentistry. The objective of this article is to summarize and discuss policies, guidelines, and relevant information on the use of teledentistry in various countries.

## 2. Methods

We classified included countries according to: (1) the World Bank income levels based on the gross national income (GNI) per capita in 2021 (12) into low (L), $1,085 or less; lower middle (LM), $1,086–$4,255; upper middle (UM), $4,256–$13,205; and high (H), $13,205 or more. We also reported on (2) the technology infrastructure in the 19 countries included in the study using data from the 2021 statistics of the International Telecommunication Union (13). The indicators used were (2a) the affordability of services for mobile-voice and mobile data measured as percentage of the price of these services to monthly GNI per capita in 2021. This indicator assesses countries' progress towards achieving the Broadband Commission for Sustainable Development target for 2025, of making the price of entry-level broadband services in developing countries <2% of monthly GNI/capita (14, 15). Two other indicators were also used to assess coverage of internet-based ICT services: (2b) Active mobile-broadband subscriptions/100 inhabitants and the (2c) percentage of internet users based on results from national households surveys. The denominator for this last percentage is the total population of the country; or at least individuals of 5 years age and older.

We also compared countries regarding the situation of (3) policies and programs for telemedicine or telehealth using the World Health Organization (WHO) Global Observatory 2015 data for eHealth, published in the Atlas of eHealth country profiles (16). This comparison assumed that countries with existing telehealth or telemedicine related policies and programs may be more likely to establish teledentistry policies and programs. For each country, we identified whether there were (3a) national policies or strategies for electronic health (eHealth), (3b) health information system (HIS) and (3c) telehealth. We reported on the country-level situation of programs for (4a) remote patient monitoring and (4b) mobile telehealth which were both classified regarding program level (4.i into 4.v) and type (4.1 to 4.3). Program levels included (4.i) international (health entities in other countries in the world), (4.ii) regional (health entities in countries in the same geographic region), (4.iii) national (referral hospitals, laboratories and health institutes, mainly public, but also private), (4.iv) intermediate [covering district or provincial facilities, public, private for-profit and private not-for-profit (e.g., religious) hospitals and health centres] and (4.v) local or peripheral (health posts, health centres providing basic level of care). Program types were divided into (4.1) informal (use of ICT for health purposes in the absence of formal processes and policies), (4.2) pilot (testing and evaluating a program) and (4.3) established (an ongoing program that has been conducted for a minimum of 2 years and is planned to continue).

We scanned the literature about teledentistry in the last 1–2 years and identified researchers publishing about teledentistry in their countries and they were contacted by the core study team to invite them to participate. The researchers from these different countries were invited to provide an overview of the status of implementation of teledentisry in their countries before and during the COVID-19 pandemic. The researchers identified laws, policies and statutory regulations governing the practice of teledentistry in their countries. They provided an overview of teledentistry program levels, types, providers and regulators. The researchers gave an overview of country-specific scientific publications, websites, national records, and reports on the impact and challenges associated with the use of teledentistry in their respective countries. This was done based on their firsthand knowledge of their country situation, main stakeholders and information sources. No systematic search strategy was used since the aim was to report on country situation. Country data were categorized by region and presented alphabetically.

## 3. Results

### 3.1. General overview

Table 1 shows the national policies for eHealth, HIS and telehealth, the ICT infrastructure affordability and coverage, as well as the type and level of remote patient monitoring and mobile telehealth programs in each of the 19 countries included in the study. Most (10, 52.6%) countries were high income, five (26.3%) were upper middle income and four (21.1%) were lower middle income. Most (11, 57.9%) countries have an existing eHealth policy, 7 (36.8%) have HIS policy and five (26.3%) have telehealth policy. The cost of mobile data and low-consumption voice services in five countries (Bosnia and Herzegovina, Libya, Nigeria, South Africa and Zimbabwe) was higher than the 2% target set for 2025. Also, most countries had higher rate of active mobile broadband subscription and internet users than the global average of 78.9% and 69.8% respectively. The greatest number of remote patient monitoring programs were at local (*n* = 6) or intermediate (*n* = 5) levels and pilot (*n* = 7). Also, the greatest number of mobile telehealth programs were at the local level (*n* = 7) and pilot (*n* = 5). There were no national remote patient monitoring programs in any of the included countries. Only Finland had a regional mobile telehealth program whereas Canada and Chile had national mobile telehealth programs. Established programs for remote patient monitoring (*n* = 4) or mobile telehealth (*n* = 4) represented a minor proportion.

**Table 1:** Overview of technology infrastructure, digital health policies, telemedicine and teledentistry programs in the 19 countries.

Country	Income	eHealth policy	HIS policy	Telehealth policy	Mobile data & voice low-consumption basket/GNI pc 2021 (ref)	Active mobile-broadband subscriptions/100 inhabitants 2021 (ref)	Internet users (%) 2021 (ref)	Remote patient monitoring program, level & type	Mobile telehealth program type	Policy/strategy regulating teledentistry	Teledentistry program
**Europe**											
Bosnia & Herzegovina	UM	N	N	N	2.03	56.1	75.7	–	–	N	N
Finland^a^	H	Y	Y	Y	0.53	157.2	92.8	Local/pilot	Regional, local/established	Y	Intermediate/established
France	H	–	–	Y	0.59	100.4	86.1	–	–	Y	Intermediate/established
Italy	H	Y	Y	Y	0.64	96.8	74.9	Intermediate/established	Local/informal	N	Local/informal
UK	H	Y	Y	Y	0.39	113.3	96.7	Intermediate/established	Intermediate/informal	N	National/pilot
**The Americas**											
Canada	H	Y	N	N	1.91	75.1	92.8	Intermediate, local/pilot, established	National, intermediate, local/pilot, established	Partly	Intermediate/informal
Chile^a^	H	Y	Y	N	0.62	110.8	90.2	Intermediate/established	National/established	Y	National/pilot
Mexico	UM	N	Y	N	1.39	85.9	75.6	Local/pilot	Local/pilot	N	Local/informal
**Africa**											
Egypt	LM	–	–	–	0.78	61.4	72.1	–	–	N	Local/informal
Libya	UM	–	–	–	2.28	17.0	17.8^b^	–	–	N	Local/informal
Nigeria	LM	–	–	–	3.33	36.6	55.4	–	–	N	N
South Africa	UM	Y	Y	N	2.43	115.7	100	–	Local/pilot	N	Local/informal
Zimbabwe	LM	Y	Y	N	12.58	58.3	34.8	Local/pilot	–/informal, established	N	National/pilot
**Asia and the pacific region**											
China	UM	Y	N	Y	0.68	101.6	73.1	Local/pilot	Local/pilot	Y	Intermediate/established
Hong Kong	H	–	–	–	0.18	160.3	93.1	–	–	N	Local/informal
Iran	LM	Y	N	N	NA	104.5	78.6	Intermediate/pilot	Intermediate/informal	N	Local/informal
New Zealand	H	Y	N	N	0.31	95.4	95.9	Local/pilot	Local/pilot	Y	Local/informal
Qatar	H	Y	N	N	0.4	144	100	–	–	N	National/pilot
Saudi Arabia	H	–	–	–	0.74	119.5	72.3	–	–	Y	National/pilot
Global		58%	66%	22%	15.0	78.9	69.8	–	–	–	–

Income levels: LM, lower middle ($1,086–$4,255 GNI per capita in 2021); UM, upper middle ($4,256–$13,205 GNI per capita in 2021); H, high ($13,205 or more GNI per capita in 2021); Regional level: Health entities in countries in the same geographic region; National level: Referral hospitals, laboratories and health institutes (mainly public, but also private); Intermediate level: District or provincial facilities: public and private hospitals and health centres; Local or peripheral level: Health posts, health centres providing basic level of care Informal: Use of ICT for health purposes in the absence of formal processes and policies; Pilot: Testing and evaluating a program; Established: An ongoing program that has been conducted for a minimum of 2 years and is planned to continue.

^a^ Data updated by personal communication with authorities in the Ministry of Health.

^b^ ITU estimate for 2014 which is the most recent.

Based on the country reports of teledentistry programs that will be presented in the next section, two countries, Italy and the UK, had telehealth policies but no specific teledentistry policy or strategy. Also, Chile, New Zealand and Saudi Arabia have teledentistry policies or strategies although, based on the WHO 2015 statistics (16), they had no policy or strategy for telehealth. France has an established teledentistry program applied at the intermediate level but no report of a similar telemedicine program and so did Qatar and Saudi Arabia whose teledentistry programs were piloted at national levels during the COVID-19 pandemic. In addition, informal teledentistry programs were tried at local levels in Egypt, Hong Kong, and Libya during the COVID-19 pandemic with no previous history of mobile telehealth programs documented by the WHO in 2015 (16).

### Country wise teledentistry programs

#### 3.2. Teledentistry in Europe

The European Commission has been developing a governance framework to promote better use of data for healthcare, research, innovation and policy-making and the supporting infrastructure for all European Union member states (17). Also, the European Union formulated a financing plan as part of the *Next Generation EU* program in July 2020 (18) and some governments are using these funds to restructure healthcare services including telehealth programs.

##### 3.2.1. Bosnia and Herzegovina

###### 3.2.1.1. Policies and regulations

Bosnia and Herzegovina includes two federal entities: the Republic of Srpska and the Federation of Bosnia and Herzegovina and the Brcko District. Telemedicine has been recently introduced in the Law on Healthcare of the Republic of Srpska but is not addressed in the laws of the Federation of Bosnia and Herzegovina and Brcko District. Currently, there are no national telemedicine, teledentistry, or E-health policies or strategies, nor HIS policy or strategy in Bosnia and Herzegovina.

###### 3.2.1.2. Projects

Electronic health record and E-prescription are used, based on entities' bylaws (19). There are no teledentistry programs at the national, intermediate or local levels. There is, however, one platform using telemedicine for online consultations with healthcare professionals (20).

#### 3.2.2. Finland

##### 3.2.2.1. Policies and regulation

In conformance with the EU regional policies, the National Patient Data Repository (Kanta) was developed in Finland for archiving electronic patient data in healthcare services (21), and the e-Prescription systems were developed to ensure that all prescriptions were issued electronically. These processes are regulated by the Act on the Electronic Processing of Client Data in Healthcare and Social Welfare which came into force in November 2021. The Finnish municipal health centres are independent and can obtain their electronic Health Information Systems (HIS) based on the municipalities' needs. Currently, there are six HIS utilised by the oral healthcare sector in different municipalities in Finland. The Association for Finnish Municipalities evaluates the different HIS to encourage the compatibility of systems among regions.

The Finnish Society of Telemedicine and eHealth (FSTeH) promotes the use of ICT in healthcare in Finland and internationally (22). The FSTeH, in collaboration with the Finnish Medical Association, the Finnish Dental Association and the Finnish Veterinary Association, coordinates a special competence degree offered to physicians, dentists, and veterinarians on eHealth (23). In addition, training on digital health services has been incorporated into the medical and dental education in line with guidelines of the European Medical Associations (24, 25).

##### 3.2.2.2. Projects

Dental offices utilize HIS to share information between places of care (26). During the COVID-19 pandemic, several Finnish public health providers and health centres offered telecommuting or remote work services including specialist consultations, video meetings, specialist and staff education, evaluation of treatment need, orthodontic controls, health promotion and preventive dental care and self-care information (27). The most recent survey showed that teledentistry programs were established at intermediate level with 22% of Finnish dentists using mobile applications in patient communications, 18% for running errands, and 13% for remote consultations with digital data transfer (28).

#### 3.2.3. France

##### 3.2.3.1. Policies and regulations

The French law (29) defines telemedicine as *a remote medical activity using ICT, to connect a medical professional with one or several health professionals or with a patient*. Thank to this law, dentists, who are included in the definition of medical professionals, are allowed to practice teledentistry, including teleconsultation, teleexpertise, telemonitoring, medical help online and medical emergency regulation (30). Telehealth must follow the same regulation as face-to-face practice including information provision, consent, and privacy (31, 32). The French national health agency began reimbursing medical doctors for limited telemedicine services in 2018 and for all telemedicine services since the COVID-19 pandemic. However, dentists are not yet paid for teledentistry services.

##### 3.2.3.2. Projects

In 2014, teledentistry started in France in Montpellier with the e-DENT project (33) which was supported by the regional health agency. The program offered asynchronous dental consultation remotely for disabled people (34), elderly people and prison inmates (35, 36). In the program, health professionals record dental and other data using an intra-oral camera and a specific software and send them to a remote dentist to diagnose and propose a treatment plan. More than 10,000 procedures were provided by the Teledentistry Department at the University Hospital of Montpellier since this program was established. During the COVID-19 pandemic, the use of teledentistry decreased possibly because oral health was not a priority and due to concerns about infection. The e-DENT project was cited by the Ministry of Health (37) and the World Health Organization (1) as a model of teledentistry and has been implemented in other countries like Singapore. In addition, there are other teledentistry programs in France including telepathology for the elderly (38), synchronous dental consultations (39) and mobile applications by which patients send photos captured by smartphone to their dentists and by which orthodontic procedures are monitored.

#### 3.2.4. Italy

##### 3.2.4.1. Policies and regulations

Using loans from the EU Next Generation program, the Italian government developed the *National Recovery and Resilience Plan* (NRRP), with investments to modernize the country. NPRR Mission 6 has an ambitious telemedicine program for territorial healthcare assistance and digitalization of the national health service. The *National Agency for Regional Healthcare Services* supported by the *Digital Transformation Department* set guidelines for regional projects of telemedicine services to achieve the goals of Mission 6 (40). There are, however, no laws regulating the practice of teledentistry in Italy at the present time.

##### 3.2.4.2. Projects

The COVID-19 pandemic highlighted the need for the harmonization of guidelines and application models to transition from experimentation to widespread adoption of telehealth services in Italy. The pandemic made it clear that digital tools are important and that healthcare provision through local areas is more needed than through central hospitals. Teledentistry facilitated the remote management of dental emergencies during the COVID-19 pandemic (41, 42). The guidelines for managing orthodontic patients published by the Italian Orthodontic Society promoted the use of photographic documentation or video calls to identify patients who needed in-person or remote care (43).

##### 3.2.4.3. Barriers

Several aspects, however, still need to be regulated including privacy, data protection, informed consent and technical and organizational measures to avoid data loss throughout the service cycle.

#### 3.2.5. United Kingdom

##### 3.2.5.1. Policies and regulations

Teledentistry was occasionally used in the UK before 2020, mostly for orthodontic consultations (44) with limited evidence on its value (45) and some experts worried that teledentistry may increase inequalities in access to dental care (46).

Because of the COVID-19 lockdown after March 2020, telehealth was encouraged by the National Health Service (NHS) to maintain communication with patients.

##### 3.2.5.2. Projects

The standard operating procedures for emergency dental care in the UK recommended remote risk assessment and triage to manage patients with urgent dental problems (47). At that time, a survey showed that 75% of dental care workers and trainees had no prior experience with teledentistry, 63% had no access to video consultation resources and 16% did not feel confident they can make the correct diagnosis using teledentistry (48). However, to avoid cancellation of outpatients' appointments in hospitals, virtual care was introduced to replace face-to face dental appointments (49). The NHS initiated the nationwide *Attend Anywhere* service, a web-based platform where pre-arranged video consultations were used to link dental healthcare workers and patients (49).

Also, the lockdown offered an opportunity for utilizing teledentistry to train dental students. These opportunities improved dental care providers' confidence in the value of consultation and validity of diagnoses made by teledentistry (45). In surveys conducted after the pandemic, patients, dentists and dental students indicated their satisfaction with teledentistry (45, 49). Patients also reported that teledentistry reduced the anxiety they experienced with traditional face-to-face consultations (45).

##### 3.2.5.3. Barriers and facilitators

Given the satisfaction with teledentistry in the UK during the pandemic, it is likely that dental health care providers will continue to use it in the future, although currently, no specific law regulates this practice.

### 3.3. Teledentistry in the americas

#### 3.3.1. Canada

##### 3.3.1.1. Policies ad regulations

Teledentistry has been of interest to Canadian oral health policy makers before COVID-19 as an auxiliary tool to deliver care to populations with limited access to care, such as hospitalized patients, residents in senior homes, special needs patients, and people in remote areas (50). However, in contrast to telemedicine, guidelines for teledentistry were sparse. The closure of dental clinics during COVID-19 magnified the need for virtual care and digital dental care services despite the absence of guidelines to manage the risk to privacy, remote informed consent process, record keeping, insurance coverage and billing (51).

To address these gaps, regulatory provincial bodies developed guidelines for teledentistry including asynchronous/store and forward or synchronous/live modalities, thereby setting standards for patients' virtual consent, handling personal information, and ensuring that teledentistry is aligned with best practices for dental care.

##### 3.3.1.2. Projects

Most Canadian jurisdictions recommended the triage of dental emergencies via teledentistry (52). In addition, the Canadian Dental Association identified codes and associated fees for the provision of services through teledentistry (53).

##### 3.3.1.3. Barriers and facilitators

Despite these rapid advances, there are knowledge gaps about the magnitude of teledentistry implementation in Canada. For example, the proportion of dental professionals currently using teledentistry codes for triage, consultations, oral examinations, and referrals remains unknown. Also, information is insufficient on whether dentists can bill for dental services offered virtually in the absence of updated information about the new teledentistry codes in fee guides at insurance companies (51). Meanwhile, teledentistry modules have been incorporated in the curricula of dental students to equip newly graduating dentists with the needed training (54).

#### 3.3.2. Chile

##### 3.3.2.1. Policies and regulations

Teledentistry practice was framed within the 2014–2018 Chilean National Digital Health Strategy (CNDHS) (55). The strategy aimed to optimize the role of specialised human resources and improve access to healthcare, especially for communities far from urban centres or those with problems of specialists' supply.

The national telehealth policies in Chile provide opportunities for the practice of teledentistry in the public service through the implementation of the telehealth policy. The policy supports the development of infrastructure, reimbursement of telehealth services and establishment of Digital Hospital that provides telemedicine, teleducation and teleassistance since 2018 (55).

##### 3.3.2.2. Projects

During the COVID-19 pandemic, teledentistry enabled dental professionals to continue to provide care to patients. Programs like oral telepathology and tele-orthodontics started before the COVID-19 pandemic such as (56). During the pandemic, telepathology provided 549 public health consultations throughout Chile (57) and dental emergency triage was proposed (58).

Currently, there is a dedicated national program for teledentistry that provides consultations, triage, and monitoring for oral pathology, orthodontics, temporomandibular disorders, and oral surgery.

##### 3.3.2.3. Barriers

There are currently no regulations specifically governing the practice of teledentistry, which makes it difficult for dental professionals to know what the boundaries of practices are. Additionally, telehealth infrastructure and reimbursement mechanisms vary among regions, creating challenges for the use of teledentistry outside of the public dental health sector.

#### 3.3.3. Mexico

##### 3.3.3.1. Projects

There are some mobile healthcare initiatives in Mexico that are led by national institutions such as the National Institute of Public Health (INSP), public universities such as the University of Veracruz, private universities such as Instituto Tecnológico de Estudios Superiores de Monterrey and private initiative such as the Carlos Slim Foundation. These initiatives are based on the use of SMS messaging and applications to provide care for some diseases that do not include oral health (59).

##### 3.3.3.2. Barriers

There are barriers to provide teledentistry services that limit its generalization to the whole population in Mexico. These barriers include income inequalities which may impede the possibilities of equitable access to and use of teledentistry. The barriers also include limited internet coverage and low digital illiteracy (60). Although the development of a legal framework for teledentistry was proposed as part of the Telehealth Bill of 2015 (61), a lot still needs to be done about the care and management of data using various means of communication to ensure the national implementation of teledentistry.

### 3.4. Teledentistry in Africa

#### 3.4.1. Egypt

##### 3.4.1.1. Policies and regulations

There are no specific regulations governing Teledentistry in Egypt. The Personal Data Protection Law #151 for 2020 (62) stipulates that healthcare providers must apply the same standards of in-person consultations to telehealth appointments to ensure that patients receive the best care. It posits that the electronic processing of personal data is allowed if the data subject consents to this processing or if the processing is for benefit of the data subject.

##### 3.4.1.2. Projects

In September 2021, the government piloted a telediagnosis initiative in 150 medical units to be increased to 300 units. This project is a joint collaboration among the ministries of health, higher education and communication and aims to extend this service to 120 university hospitals including dental hospitals (63). In 2022, the General Authority for Healthcare Accreditation and Regulation (GAHAR) announced its collaboration with the World Health Organization, the Ministry of Health and the Supreme Council of University Hospitals to establish a telehealth law followed by GAHAR criteria for telehealth in the coming period (64).

During the COVID-19 pandemic, there was an increase in the demand for remote dental consultations through phone calls and 50% of endodontists reported that phone calls replaced some in-office visits (65). Also, a health insurance company, GlobeMed Egypt, launched the first telehealth consultations services in Egypt. The company offers insured members access to dental care through a list of dentists. Members request a consultation through Whatsapp, get confirmation within 24 h and connect with the dentist (66).

##### 3.4.1.3. Barriers

The absence of regulations, the limited platforms for virtual delivery of dental consultations and the high cost of specialized digital equipment are challenges facing the broad implementation of teledentistry in Egypt (65).

#### 3.4.2. Libya

##### 3.4.2.1. Policies and regulations

Based on the recommendations of the National Center for Disease Control in Libya and the Libyan Dental Association, most Libyan dental offices closed in April 2020, because of concerns about patient safety and COVID-19 transmission (67). The Libyan government, particularly the health ministry, established laws and regulations to improve the digitization of healthcare. This is especially important in rural areas where specialists may not be easily accessible to patients.

##### 3.4.2.2. Projects

Through agreements between academic hospitals in Libya and other countries, telemedicine technologies allowed consultations for diagnosis and treatment as well as participation in Libyan medical conferences. Some examples of teledentistry during the pandemic include Libyan dental practitioners using WhatsApp to differentiate between oral lesions by dental photography because many oral lesions are readily visible, thus reducing the need for direct clinical assessment (68). Through an aid project with the European Union, Libya established a teledentistry project among five universities (University of Tripoli, University of Zawia, University of Sirte, University of Misurata, University of Sebha, and Libyan International Medical University) to fund and supply the required equipment thus helping teledentistry application in Libya (69, 70).

##### 3.4.2.3. Barriers

There are technical and infrastructure challenges to teledentistry in Libya, including interrupted internet service, lack of hardware, and lack of training and technical guidance particularly in rural areas, which remain unconnected by various mobile service providers. The challenges also include the acceptance of teledentistry by dental professionals and patients who need training and education to remove this barrier.

#### 3.4.3. Nigeria

##### 3.4.3.1. Policies and regulations

There are neither specific laws nor agencies regulating telemedicine in Nigeria though there was an attempt to develop an e-health policy in 2011 (71). The National Information Technology Development Agency (NITDA) monitors the use of electronic data and, in 2019, issued the Nigeria Data Protection Regulation (NDPR) for the exchange of personal data (72). There are, however, some loopholes in the provisions by the NITDA on data retention and the liability of third parties managing telemedicine especially foreign partners. The transfer of data to a foreign country should be under the supervision of the Attorney General of the Federation to allow regulatory agencies to impose appropriate penalties in case of non-compliance with the regulations (73). Also, the current guidelines regulating e-health do not protect the security of health-related personal data nor the cycle of data collection, processing, retention and finally, deletion (73).

##### 3.4.3.2. Projects

The COVID-19 pandemic accelerated the practice of telemedicine with 400% increase in the use of Hudibia, a Nigerian telemedicine platform (74). During the pandemic, 90% of healthcare professionals used telemedicine to deliver health care, and 63% of consumers received healthcare through telemedicine (75). However, 68.33% of dental health care practitioners were not aware of teledentistry and no dental healthcare practitioner ever used the services (76). There is also no evidence of introducing teledentistry in the training curriculum of students. This is a competency that needs to be developed by training new dental healthcare personal. With 1.4 thousand dentists serving 206 million people in Nigeria (77), teledentistry has the potential to address inequalities in access to oral health care.

##### 3.4.3.3. Barriers

Currently, there are no guidelines regulating teledentistry. The multiple dental practice and dental professional regulatory agencies need to fast-track the institution of tele-dentistry practice in Nigeria.

#### 3.4.4. South Africa

##### 3.4.4.1. Policies and regulations

Guidelines for telemedicine were established by the Health Professions Council of South Africa (HPCSA), the national health regulatory body, although the practice was limited to online consultations between medical professionals only (78, 79). These guidelines were used for teledentistry and researchers discussed their applicability years before the COVID-19 pandemic (80). The COVID-19 pandemic renewed the interest in teledentistry, although it was not used during this period (81, 82).

##### 3.4.4.2. Barriers and facilitators

Some of the challenges identified with implementing teledentistry in South Africa include legal issues regulating health care provision and dentist- patient relationship, ethical considerations related to consent and assent for minors, confidentiality, security and protection of personal information and the need to establish a system to refer patients to practitioners and specialists. There was also the issue of the type of teledentistry platforms and how appropriate they were to the local context, and the remuneration models provided by healthcare funders to ensure a sustainable system for funders, service providers and users (81–83).

It is expected that the Oral Health Center of South Africa, the largest in the continent and the only one accredited by the World Health Organization as a collaborating centre, will address the use of mobile technology and teledentistry which are priorities in its agenda (84). The research at this institution together with evidence created by the three other dental faculties in South Africa will facilitate the incorporation of teledentistry in the national oral health policy in the teachings at tertiary level and in practice in the private and public sector (85).

#### 3.4.5. Zimbabwe

##### 3.4.5.1. Policies and regulations

The Medical and Dental Practitioners Council of Zimbabwe held a position that telehealth services were prohibited (86, 87) and the Health Professions Authority dissuaded members of the public from using telehealth services (88) because telehealth services were not supported by law and violated the Health Professions Act which requires the registration of any health premise. There were also ethical concerns about the potential for telemedicine and teledentistry to delay the examination and treatment of patients.

##### 3.4.5.2. Projects

In 2018, an oral health Facebook page was established to increase oral health awareness and build momentum for the World Oral Health Day celebrations. Also, Solidarmed Zimbabwe introduced a multidisciplinary, countrywide eHealth project to support HIV, tuberculosis, health for mothers and newborns and non-communicable diseases programs for rural populations and achieve universal health coverage (89).

The COVID-19 pandemic fast-tracked the use of virtual platforms to offer health services to the public (90) and health education and digital tools to health professionals (91–93). With the closure of dental practices to contain the pandemic, the Medical and Dental Practitioners Council allowed healthcare practitioners to use telemedicine and teledentistry and charge for their services. Practitioners were, however, encouraged to advise patients to seek face-to-face consultation when there were doubts with medical diagnosis (94). The Ministry of Health and Childcare has drafted the Digital Health Strategy 2021–2025 to promote digitization of health services delivery and enable Zimbabwe to meet its Universal Health Coverage obligation before 2030 (95). In 2021, the government rolled out a pilot telemedicine program to overcome barriers to healthcare delivery in remote areas (96).

##### 3.4.5.3. Barriers

There are concerns over data protection, funding, and standardisation of ehealth practices and the need for more stringent laws and regulations on the full coverage practice of telehealth (97–99).

### 3.5. Teledentistry in Asia and the pacific region

#### 3.5.1. China

##### 3.5.1.1. Policies and regulations

In China, dentists are physicians specializing in oral diseases (stomatologists) and dentistry is a discipline within medicine (100). Thus, instead of teledentistry programs, the use of ICT for oral healthcare is under telemedicine programs. China has begun to explore tele healthcare since the 2010s, with consecutive government directives being issued to guide the process (101, 102). By 2019, and before the COVID-19 pandemic, 74% of region-level or tertiary hospitals and 56% of city-level or secondary hospitals had already adopted some sort of tele-consultations, although remote treatment was relatively rare (103).

##### 3.5.1.2. Projects

Dental professionals began the first teledentistry project in 2012 through a joint remote consultation among Peking University Stomatological Hospital, Fourth Affiliated Hospital of Guangzhou Medical University, Dalian Stomatological Hospital, QingDao Stomatological Hospital and Zunyi Medical College Affiliated Stomatological Hospital (104).

During the COVID-19 pandemic, China accelerated the adoption of ICT into healthcare to facilitate remote treatment with emphasis on emergency care, remote diagnosis, remote treatment and remote intensive care (105). In February 2020, the Hospital of Stomatology Wuhan University started the first local teledentistry service for the remote diagnosis of Wuhan residents, focusing on identifying potential causes of oral diseases and instructing patients to relieve symptoms through self-care (106). Such services expanded to other cities, and in some regions evolved into remote restorative treatments for remote settlements and border garrisons (107, 108). Recently, Fujian Medical University Affiliated Stomatological Hospital reported that its team had performed the very first remote dental implant treatment in China by controlling surgery robots with 5G networks (109).

#### 3.5.2. Hong Kong special administrative region, China

##### 3.5.2.1. Policies and regulations

Since 2017 Hong Kong Government has proposed the idea of smart city, which applies technology in daily life including transportation, “Smart Mobility”, living, “Smart Living”, and environment, “Smart Environment”, with “Smart People”, “Smart Government” and “Smart Economy”. However, there were only minor attention to smart technology planning and policy in medicine and dentistry (110). There is currently no significant public initiative to promote tele-healthcare/tele-dentistry, and there are no related guidelines or regulations on the privacy of remote healthcare, though in “smart city”, plans were mentioned to explore telehealth, videoconferencing and remote consultation in Hong Kong (110).

##### 3.5.2.2. Projects

During the COVID-19 pandemic, tele-medical consultations were used although, most were not related to dentistry (111). In Hong Kong, dentistry is run by the private sector unlike medicine, and the government does not provide large scale public dental services. The cost of setting up tele-consultation is too high for the dental private sector. Also, intraoral photograph and radiographs are needed for diagnosis and these further require special equipment that preclude tele-consultation. Orthodontics is an exception where there are clear aligner orthodontic appliances with some tele-consultations (112) where patients can consult dentists from other countries. Some smartphone applications or websites provide oral health education using multimedia such as videos, animation, figures and text.

The Faculty of Dentistry, University of Hong Kong, is exploring the deployment of teledentistry in Hong Kong. There are trials running currently on an AI-powered smartphone photography that would allow users to detect gum health (113) and an AI-based web tool to predict oral cancer risk, the Deepsurv (114).

#### 3.5.3. Iran

##### 3.5.3.1. Policies and regulations

There are no specific policies governing the practice of teledentistry in Iran.

##### 3.5.3.2. Projects

Remote dental services provided in Iran include specialized telephone or video consultation, tele-triage, remote examination, screening of dental patients for COVID-19 symptoms, patient education and monitoring, e-prescription, remote follow-up of treatment and management and dental health education. The infrastructure used to deliver teledentistry services include internet, telephone, smartphone, video call, SMS, email, central database, electronic prescribing, electronic patient record, Dental Information Systems, picture archiving and communication system (PACS) and educational platforms. The services might be provided as synchronous or asynchronous teledentistry. Asynchronous teledentistry includes transmission of recorded health information such as radiographs or digital impressions by a practitioner to send to specialized laboratories to provide aligners in orthodontic treatments or bridge and crowns in CAD-CAM technology in prosthodontics and cone beam CT scans to create a custom surgical guide for implant placement.

The practice of teledentistry is enhanced by the increasing use of mobile phones. As high as 90% of Iranians used mobile phones in 2017 and the total number of mobile cellular subscription in 2020 was 127 million for 87.29 million population (115, 116) and this number increased during the COVID-19 pandemic (117). There is also an increasing trend of using mobile devices such as smart phones or tablets for patient oral health education as part of research work with applications becoming later available on Bazaar (the Iranian app market) free or at a low cost.

##### 3.5.3.3. Barriers and facilitators

Though teledentistry may help solve some of the access to dental healthcare problems especially in remote deprived areas where there is shortage of skilled labor and limited financial resources (118), there are still problems to contend with. These include the low internet speed in synchronous procedures and the lack of policies and agencies regulating the practice of ehealth. Teledentistry is currently supervised by the Ministry of Health, the Iranian Dental Association, and the Telemedicine Association in Iran.

#### 3.5.4. New Zealand

##### 3.5.4.1. Policies and regulations

Teledentistry practice in New Zealand is regulated by laws (119) implemented by the Dental Council of New Zealand that serves as the regulatory authority (120). It provides information and advice on Teledentistry (121). Teledentistry is considered an adjunct to face-to-face clinical care and cannot serve as a substitute (122). The guidelines require that patients are screened to identify the need for teledental consultation vs. face-to-face visit (119). Also, clinicians should not put the patient's health or safety at risk. If the clinician cannot apply quality standards, or teledentistry cannot meet the patient's needs, the patient should be referred to where they can receive the care they need (121).

##### 3.5.4.2. Projects

During teledental consultation, clinicians follow standard procedures including informed consent. Patient's identity and personal details must be verified and any health personnel present in the virtual consultation room must be introduced before the session to ensure that patient's privacy is protected. Patient records should be accurately documented and stored in a secure computer system with authorized software, firewalls, anti-malware and a backup system. The virtual platforms commonly used for teledentistry in New Zealand include Google Meet, Microsoft Teams, Cisco Webex, and Zoom. Teledentistry has been widely used in New Zealand since the COVID-19 pandemic (122, 123) for triaging to determine whether treatment is urgently needed or could be delayed (123).

##### 3.5.4.3. Facilitators

Teledentistry seems to be well-accepted in New Zealand although patients with chronic pain were less satisfied with treatment provided virtually than those without pain. Nevertheless, patients and clinicians evaluated teledentistry positively, and were comfortable with the procedures and accurate diagnosis obtained (122).

#### 3.5.5. Qatar

##### 3.5.5.1. Policies and regulations

The Qatari Law for Healthcare Provision (#22/2021) (124) guarantees the confidentiality of beneficiaries' health data and lays penalties for confidentiality breaches but does not specifically address telehealth or teledentistry practices.

##### 3.5.5.2. Projects

During the pandemic, the government established a hotline to support access to oral health care in 2020 (125). All hotline teledentists were trained, had dedicated workspaces for caller privacy and were required to complete a teledentistry data form for each call. Special algorithms were designed to categorize triage levels to assist hotline dentists in managing self-reported complaints (126). The algorithms enabled teledentists to arrive at a diagnosis and decide on who should receive face-to-face dental treatment including emergencies or remote care with courier deliveries of medication. In face-to-face treatment, protocols were used to classify procedures into non-aerosol generating procedures (low risk) and aerosol generating procedures (high risk). Till 2022, 70.6% of callers were non-urgent dental cases. All non-urgent cases were treated remotely; 80% needed only advice and 20% needed medication. Care was provided to the elderly, people with physical disabilities, people in remote locations, and those with dental phobia (126).

##### 3.5.5.3. Barriers

There are several challenges facing teledentistry in Qatar. These include inadequate training of dentists, legal issues pertaining to patient consent; privacy and confidentiality of computer-based patient data; potential for inaccurate diagnosis resulting from inability to assess lesions or teeth through palpation or vitality tests; patient technology illiteracy; requirements for sophisticated workstations; and patient awareness of teledentistry.

#### 3.5.6. Saudi Arabia

##### 3.5.6.1. Policies and regulations

In 2021, the Saudi Ministry of Health (MOH) developed an e-health strategy in collaboration with national and international advisors and ICT companies such as IBM (127). The National Health Information Center regulates the exchange of health data electronically between health sectors (128).

##### 3.5.6.2. Projects

In 2022, the MOH established “Seha or Health” App, the largest virtual hospital worldwide (129), to offer telehealth consultations (130). The Ministry works with the private sector such as Philips (131) and Apple (132) to provide teledentistry consultations to patients and options for monitoring and follow up to dentists.

Teledentistry has been widely used in Saudi Arabia during the COVID-19 pandemic for virtual consultations (11, 133) through social media such as Twitter, WhatsApp and Facebook (11, 133) especially for orthodontic care (134). Teledentistry was particularly useful in remote areas with dentists' shortage and limited dental facilities (11). Teledentistry was also used to disseminate audio-visual oral health information, using applications such as Skype, Microsoft Teams and Zoom (11, 135–137); for referral (136); appointments scheduling (138); and case discussion between specialists and general practitioners (136, 137).

##### 3.5.6.3. Barriers and facilitators

The public perception about teledentistry was positive. Users indicated that teledentistry helped reduce the cost of access to care (136), was reliable in caries detection among children (139) and improved mothers' knowledge and awareness of the oral health needs of their children (140).

Concerns were raised that dental practitioners had limited knowledge of teledentistry and its application (11, 133, 141) and some practitioners had little confidence in sharing patients' documents online (137) due to concerns over patients' privacy (9, 137, 141). In addition, not all patients can use online applications or have internet access, and teledentistry cannot help patients who need dental treatment procedures (9, 133).

## 4. Discussion

This current study provides information on the status of implementation of teledentistry in 19 countries from several regions. The findings indicate that most countries do not have laws, policies or regulatory bodies governing telehealth, and where there were policies on telehealth, many do not have policies governing teledentistry specifically. In most cases, teledentistry is implemented at local level as an informal project. The practice of teledentistry however, gained momentum during the COVID-19 pandemic, and this may facilitate its integration into national oral health policies, guidelines and practices where it is not yet implemented.

The study fills a knowledge gap on the status of implementing teledentistry, and the possible impact of COVID-19 on establishing systems to support its practice in-country. We profiled the practice of teledentistry in several countries from different regions with different political, technological, economic and healthcare system structure. One of the limitations of the study is the limited number of countries that represent the regions. We tried to overcome this limitation by providing an overview of regional policies governing the practice of ehealth, and by reflecting on the impact of these regional policies on country level practice of teledentistry. Another limitation is the quality of evidence on problems and solutions facing the implementation of teledentistry in different countries. This calls for further research in this area to better understand the barriers and facilitators to implementation. Despite these limitations, the study has several important findings.

First, it appears that the availability of teledentistry policies and laws at country level facilitates the implementation of teledentistry. For example, Finland and Saudi Arabia have teledentistry policies or strategies allowing Finland to establish a teledentistry program at the intermediate level while Saudi Arabia is piloting a national teledentistry program. However, the absence of national policies does not preclude the implementation of local teledentistry practices as in Italy.

Second, some countries adapt telemedicine policies to teledentistry. However, telemedicine and teledentistry are different in scope and treatment end points. Provision of care through teledentistry is more limited than telemedicine because of the surgical approach adopted in dental care. This may explain why monitoring of orthodontic treatment, triage, prescription of medication and referral constitute a major portion of teledentistry services. Advancements in the field of robotics my introduce the second generation of teledentistry by allowing indirect interventions to be remotely provided using the internet similar to what was reported in China.

Third, in some cases, establishing telehealth policies and systems was driven by the government's commitment to follow regional policies such as in Finland where the European Union governance framework was the driving force behind establishing HIS and telehealth programs before the pandemic. In other countries, such as Chile, the driving force was the national digital health strategy developed before the pandemic in response to national needs. In most other countries, however, the COVID-19 pandemic fast-tracked the implementation of teledentistry leading to the establishment of policies, regulatory bodies and programs.

Fourth, technology infrastructure is important to launch a teledentistry program. Mobile data affordability and internet coverage seem to be associated with the presence of teledentistry programs at national or intermediate level. Chile, Finland, Qatar and the UK have mobile data and voice services at costs that are less than 1% of the gross national income and internet coverage for >90% of the population. Chile and Finland have teledentistry programs at the intermediate level while Qatar and UK are piloting national programs. This finding indicates that affordable connectivity may facilitate the establishment of teledentistry programs. Affordable connectivity is not an absolute barrier against setting up teledentistry program as indicated by the example of Zimbabwe which has the least affordable mobile data and the lowest internet coverage among the 19 countries. Yet the country hosts a local remote patient monitoring program that provides teledentistry services. Zimbabwe presents an opportunity to study the feasibility of telehealth programs in Africa where internet connectivity and cost are existing challenges; and an opportunity to learn how political will may impact the implementation of national teledentistry programs.

Fifth, different stakeholders drive teledentistry programs in different countries. The academic and research communities led the process in France and Iran launching projects and assessing stakeholders' satisfaction. In other countries, such as Canada and Finland, provincial health centers led the implementation of teledentistry programs. In Qatar, Saudi Arabia and the UK, the national governments developed the system while in Egypt and Zimbabwe, private companies operated the teledentistry systems. These variations may impact future growth and sustainability of the teledentistry systems. Research-led projects may lack sustainability after the end of the research project and are thus likely to exist at local level. National programs that are coordinated with the private sectors may have better sustainability and affordability to consumers than programs that are managed solely by the private sector.

Sixth, many factors are involved in the complex processes leading to the institutionalization of teledentistry and its incorporation it into clinical practice and healthcare systems. The presence of laws and strategies, regulatory bodies, renumeration schemes, technology infra structure (142) and training of workforce (143, 144) speed up the process that is mainly driven by political will to channel funds and assign resources to institute change even when challenges exist. Country profiles show the complex inter relations of these factors and that no one solution fits all. It is important to conduct a thorough assessment of the local context and to identify the healthcare needs that teledentistry can address so that tailored teledentistry services can be provided.

The present study shows the diversity of teledentistry programs levels, types and driving forces in different countries. The COVID-19 pandemic emphasized the impact of teledentistry on improved accessibility to oral healthcare services, especially for marginalized groups. The next phase involves institutionalizing teledentistry and its integration into national healthcare systems using implementation science with economic assessment of its value. Mapping teledentistry practices in other countries not included in this study provides a wider perspective to guide teledentistry practice.

## References

[1] World Health Organization. Mobile technologies for oral health: an implementation guide. Geneva (2021). Available at: https://www.who.int/publications/i/item/9789240035225 (Cited March 14, 2023).

[2] World Health Organization. Global strategy on digital health 2020–2025 (2021). Available at: https://apps.who.int/iris/handle/10665/344249 (Cited March 14, 2023).

[3] European Observatory on Health Systems and Policies, Fahy N Williams GA. Use of digital health tools in Europe: before, during and after COVID-19 (2021). Available at: https://apps.who.int/iris/handle/10665/345091 (Cited March 14, 2023). 35104064

[4] World Health Organization. Global diffusion of eHealth: making universal health coverage achievable: report of the third global survey on eHealth. Geneva. (2016) Available at: https://apps.who.int/iris/handle/10665/252529 (Cited March 14, 2023).

[5] Eysenbach G. What is e-health? J Med Internet Res. (2001) 3(2):1–5. 10.2196/jmir.3.1.e1 PMC1761894 11720962

[6] Oh H Rizo C Enkin M Jadad A. What is eHealth (3): a systematic review of published definitions. J Med Internet Res. (2005) 7(1):e1. 10.2196/jmir.7.1.e1 15829471 PMC1550636

[7] Eraso FE Scarfe WC Hayakawa Y Goldsmith J Farman AG. Teledentistry: protocols for the transmission of digitized radiographs of the temporomandibular joint. J Telemed Telecare. 1996;2(4):217–23. 10.1258/1357633961930103 9375062

[8] National Library of Medicine. PubMed: search-teledentistry (2023). Available at: https://pubmed.ncbi.nlm.nih.gov/?term=teledentistry&sort=date&sort_order=asc&size=200 (Cited March 15, 2023). 10.1080/15360280801989377 28792816

[9] Al Mohaya MA Almaziad MM Al-Hamad KA Mustafa M. Telemedicine among oral medicine practitioners during COVID-19 pandemic and its future impact on the specialty. Risk Manag Healthc Policy. (2021) 14:4369–78. 10.2147/RMHP.S325777 34707420 PMC8544785

[10] Hung M Lipsky MS Phuatrakoon TN Nguyen M Licari FW Unni EJ. Teledentistry implementation during the COVID-19 pandemic: scoping review. Interact J Med Res. (2022) 11(2):e39955. 10.2196/39955 35862174 PMC9307266

[11] Nassani MZ Al-Maweri SA Alsheddi A Alomran A Aldawsari MN Aljubarah A Teledentistry-knowledge, practice, and attitudes of dental practitioners in Saudi Arabia: a nationwide web-based survey. Healthc (Basel, Switzerland). (2021) 9(12):1682. 10.3390/healthcare9121682 PMC8701844 34946408

[12] World Bank. World bank country and lending groups—world bank data help desk (2023). Available at: https://datahelpdesk.worldbank.org/knowledgebase/articles/906519-world-bank-country-and-lending-groups (Cited March 15, 2023).

[13] International Telecommunication Union. Statistics (2023). Available at: https://www.itu.int/en/ITU-D/Statistics/Pages/stat/default.aspx (Cited March 15, 2023).

[14] International Telecommunication Union. ICT price baskets (IPB) (2023). Available at: https://www.itu.int/en/ITU-D/Statistics/Dashboards/Pages/IPB.aspx (Cited March 15, 2023).

[15] International Telecommunication Union. ICT prices (2023). Available at: https://www.itu.int/en/ITU-D/Statistics/Pages/ICTprices/2019default.aspx (Cited March 15, 2023).

[16] Global Observatory for eHealth. Atlas of eHealth country profiles—the use of eHealth in support of universal health coverage: based on the findings of the third global survey on eHealth 2015 (2016). Available at: https://apps.who.int/iris/bitstream/handle/10665/204523/9789241565219_eng.pdf;jsessionid=0C93AF6BC136540B1C405AA2849311F3?sequence=1 (Cited March 15, 2023).

[17] OECD/European Union. Health at a Glance: Europe 2020: state of health in the EU cycle. Paris (2020). Available at: https://www.oecd-ilibrary.org/social-issues-migration-health/health-at-a-glance-europe-2020_82129230-en (Cited March 15, 2023).

[18] Gazzetta Ufficiale della Repubblica Italiana. Approvazione delle linee guida per i servizi di telemedicina—requisiti funzionali e livelli di servizio/approval of guidelines for telemedicine services—functional requirements and service levels (2022). Available at: https://www.gazzettaufficiale.it/eli/id/2022/11/02/22A06184/sg (Cited March 15, 2023)

[19] Health Insurance Fund of Republika Srpska. Health insurance in Republika Srpska (2023). Available at: https://www.zdravstvo-srpske.org/home.html (Cited March 15, 2023).

[20] T medIT doo. Doktor Kad Mi Treba—communicate with your doctor online anytime, anywhere (2020). Available at: https://doktorkadmitreba.com/ba/ (Cited March 15, 2023).

[21] Kanta. What are the Kanta Services?—citizens—Kanta.fi (2023). Available at: https://www.kanta.fi/en/what-are-kanta-services (Cited March 15, 2023).

[22] Finnish Society of Telemedicine and eHealth. FSTeH today—STeHS (2023). Available at: https://www.telemedicine.fi/en (Cited March 15, 2023).

[23] Finnish Medical Association. Lääkäriliitto—medical education (2023). Available at: https://www.laakariliitto.fi/en/medical-education/ (Cited March 15, 2023).

[24] Levy AR Kulmala P Merenmies J Jääskeläinen J Kortekangas-Savolainen O Jääskeläinen J National MEDigi project: systematic implementation of digitalization to undergraduate medical and dental education in Finland. Finnish J EHealth EWelfare. (2019) 11(4):357–61. 10.23996/fjhw.83309

[25] Tuovinen T Reponen J Isoviita V-M Koskela T Levy A Pääkkönen J Sähköisten terveyspalveluiden opetus lääketieteessä/digital education of health services in medicine. Duodecim. (2021) 137(17):1807–13. Available at: http://hdl.handle.net/10138/349511

[26] Savola A Lahti S Salo S Huumonen S. Electronic oral health record systems in Finland—the user experiences and development needs. Finnish Dent J. (2017) 13:38–45. Available at: https://emea01.safelinks.protection.outlook.com/?url=https%3A%2F%2Fwww.lehtiluukku.fi%2Flehti%2Fhammaslaakarilehti%2F_read%2F13-2017%2F163998.html&data=05%7C01%7C%7Ccb0f7646f9034401d1f908db60722697%7C84df9e7fe9f640afb435aaaaaaaaaaaa%7C1%7C0%7C638209815603415580%7CUnknown%7CTWFpbGZsb3d8eyJWIjoiMC4wLjAwMDAiLCJQIjoiV2luMzIiLCJBTiI6Ik1haWwiLCJXVCI6Mn0%3D%7C3000%7C%7C%7C&sdata=Y20eXaybf9w%2ByPOiEdtBTnhLXOSrM6LGg5KUdjzmRFQ%3D&reserved=0.

[27] Tilander A. Corona virus: opening of telecommuting (remote work) for dentists. (Hammaslääkärin etätyölle on paikkansa.). Finnish Dent J. (2020) 27(8):6–9. Available at: https://emea01.safelinks.protection.outlook.com/?url=https%3A%2F%2Fhammaslaakarilehti.fi%2Fhammaslaakarin-etatyolle-paikkansa%2F&data=05%7C01%7C%7Cdb307603ff404566e1a808db60749a9e%7C84df9e7fe9f640afb435aaaaaaaaaaaa%7C1%7C0%7C638209826152191307%7CUnknown%7CTWFpbGZsb3d8eyJWIjoiMC4wLjAwMDAiLCJQIjoiV2luMzIiLCJBTiI6Ik1haWwiLCJXVCI6Mn0%3D%7C3000%7C%7C%7C&sdata=OqV3GKASdZ57yIeBd1L%2BC4K%2FSeFzB9982NZHunde0j0%3D&reserved=0.

[28] Finnish Dental Association. Labor market research (2022). Available at: https://www.hammaslaakariliitto.fi/fi/liiton-toiminta/tutkimukset-ja-tilastot/tutkimukset/tyomarkkinatutkimukset#.Y7P82xXMKUm (Cited March 15, 2023).

[29] French Ministry of Health and Sports. “Hôpital, patients, santé, territoires” Une loi à la croisée de nombreuses attentes/‘Hospital, patients, health, territories’ a law at the crossroads of many expectations (2009). Available at: https://sante.gouv.fr/IMG/pdf/Loi_Hpst_07-09-09.pdf

[30] French Ministry of Health and Sports. Décret n° 2010-1229 du 19 octobre 2010 relatif à la télémédecine—Légifrance/Decree No. 2010-1229 of October 19, 2010 relating to telemedicine. public heath code. p. 3 (2010). Available at: https://www.legifrance.gouv.fr/jorf/id/JORFTEXT000022932449/

[31] Giraudeau N. Teledentistry and COVID-19: be mindful of bogus “Good” Ideas! Inquiry. 58 (2021). Available at: https://pubmed.ncbi.nlm.nih.gov/33978515/

[32] Oral and Dental Telemedicine Group- French Society of Digital Health (SFSD). Recommendations for the recognition and development of oral telemedicine in France: Teleconsultation, teleexpertise and oral teleregulation/Recommandations pour la reconnaissance et le développement de la telemedecine bucco-dentaire en France: Téléconsultation, téléexpertise et télérégulation bucco-dentaire (2021). Available at: https://sfsd.fr/wp-content/uploads/2021/09/recommandations_tlmbd_sfsd.pdf (Cited March 16, 2023).

[33] Giraudeau N Valcarcel J Tassery H Levallois B Cuisinier F Tramini P Projet e-DENT: téléconsultation bucco-dentaire en EHPAD. Eur Res Telemed/La Rech Eur en Télémédecine. (2014) 3(2):51–6. 10.1016/j.eurtel.2014.04.005

[34] Olivier R Thibault D Stéphane V Aline D Camille I Jean V Oral care in facilities for disabled people: interest of teledentistry. Dent Oral Maxillofac Res. (2019) 5(4):1–4. 10.15761/DOMR.1000303

[35] Giraudeau N Inquimbert C Delafoy R Tramini P Valcarcel J Meroueh F. Teledentistry, new oral care tool for prisoners. Int J Prison Health. (2017) 13(2):124–34. 10.1108/IJPH-04-2016-0011 28581375

[36] Inquimbert C Balacianu I Huyghe N Pasdeloup J Tramini P Meroueh F Applications of teledentistry in a French inmate population: a one-year observational study. PLoS One. (2021) 16(4):e0247778. 10.1371/journal.pone.0247778 33826659 PMC8026055

[37] French Interministerial Committee for Health. Priorité prévention : rester en bonne santé tout au long de sa vie/Priority prevention stay healthy throughout your life (Press Kit) 40 (2018). Available at: https://sante.gouv.fr/systeme-de-sante/strategie-nationale-de-sante/priorite-prevention-rester-en-bonne-sante-tout-au-long-de-sa-vie-11031/ (Cited March 16, 2023)

[38] Queyroux A Saricassapian B Herzog D Müller K Herafa I Ducoux D Accuracy of teledentistry for diagnosing dental pathology using direct examination as a gold standard: results of the tel-e-dent study of older adults living in nursing homes. J Am Med Dir Assoc. (2017) 18(6):528–32. 10.1016/j.jamda.2016.12.082 28236609

[39] Doctolib. Trouvez un rendez-vous avec une dentiste/Find an appointment with a dentist (2023). Available at: https://www.doctolib.fr/ (Cited March 16, 2023).

[40] Gazzetta Ufficiale. Ulteriori misure urgenti per l’attuazione del Piano nazionale di ripresa e resilienza (PNRR)/Further urgent measures for the implementation of the National Recovery and Resilience Plan (PNRR) (2022). Available at: https://www.gazzettaufficiale.it/eli/id/2022/04/30/22G00049/sg

[41] Dalessandri D Sangalli L Tonni I Laffranchi L Bonetti S Visconti L Attitude towards telemonitoring in orthodontists and orthodontic patients. Dent J. (2021) 9(5):47. 10.3390/dj9050047 PMC8143570 33921925

[42] Maspero C Abate A Cavagnetto D El Morsi M Fama A Farronato M. Available technologies, applications and benefits of teleorthodontics. A literature review and possible applications during the COVID-19 pandemic. J Clin Med. (2020) 9(6):1–15. 10.3390/jcm9061891 PMC7356961 32560322

[43] Societa Italiana di Ortodonzia (SIDO). Indicazioni sulla gestione delle emergenze ortodontiche durante la quarantena da COVID-19/Indications on the management of orthodontic emergencies during the covid-19 quarantine. Available at: https://www.sido.it/public/media/sido-x-protocollo-ortodonzia-x-emergenza-sanitaria-da-coronavirus-italiano_def.pdf (Cited March 16, 2023).

[44] Mandall NA Quereshi U Harvey L. Teledentistry for screening new patient orthodontic referrals. Part 2: GDP perception of the referral system. Br Dent J. (2005) 199(11):727–9. 10.1038/sj.bdj.4812969 16341186

[45] Menhadji P Patel R Asimakopoulou K Quinn B Khoshkhounejad G Pasha P Patients’ and dentists’ perceptions of tele-dentistry at the time of COVID-19. A questionnaire-based study. J Dent. (2021) 113:103782. 10.1016/j.jdent.2021.103782 34400252 PMC8361006

[46] Plaza-Ruíz SP Barbosa-Liz DM Agudelo-Suárez AA. Impact of COVID-19 on the knowledge and attitudes of dentists toward teledentistry. JDR Clin Transl Res. (2021) 6(3):268–78. 10.1177/2380084421998632 33632011

[47] National Health Service. COVID-19 guidance and standard operating procedure (2020). Available at: https://www.england.nhs.uk/coronavirus/wp-content/uploads/sites/52/2020/06/C0581-covid-19-urgent-dental-care-sop-update-16-june-20-.pdf

[48] Virdee J Sharma R Ponduri S. Spotlight on teledentistry. Br Dent J. (2020) 228(11):815. 10.1038/s41415-020-1750-0 32541714 PMC7294213

[49] Rahman N Nathwani S Kandiah T. Teledentistry from a patient perspective during the coronavirus pandemic. Br Dent J. (2020) 14:1–4. 10.1038/s41415-020-1919-6 32801323 PMC7427495

[50] Emami E Kadoch N Homayounfar S Harnagea H Dupont P Giraudeau N Patient satisfaction with E-oral health care in rural and remote settings: a systematic review protocol. Syst Rev. (2017) 6(1):174. 10.1186/s13643-017-0550-3 28851449 PMC5576324

[51] Singhal S Mohapatra S Quiñonez C. Reviewing teledentistry usage in Canada during COVID-19 to determine possible future opportunities. Int J Environ Res Public Health. (2021) 19(1):31. 10.3390/ijerph19010031 35010285 PMC8751218

[52] Royal College of Dental Surgeons of Ontario. COVID-19—guidance for the use of teledentistry (2022). Available at: https://www.rcdso.org/en-ca/standards-guidelines-resources/2019-novel-coronavirus/covid-19—emergency-screening-of-dental-patients-using-teledentistry (Cited March 16, 2023).

[53] Canadian Dental Association. CDA COVID-19 Update (2020). Available at: http://www.cda-adc.ca/newsletters/covid-19/2020/april/2020-04-17-CDA-COVID-UPDATE.html (Cited March 16, 2023).

[54] Amin M Lai JY Lindauer PA McPherson K Qari H. Should dental schools adopt teledentistry in their curricula? Two viewpoints. J Dent Educ. (2021) 85(7):1238–44. 10.1002/jdd.12614 33860537

[55] Ministerio de Salud C. Crea el Departamento de Salud Digital. 595. Creation of the Department of Digital Health (2019). Available at: https://web.archive.org/web/20221212145509/https://www.portaltransparencia.cl/PortalPdT/documents/10179/62801/Resolucion+que+crea+HD+en+GABREDES.pdf/4758cfea-6e31-4ca2-985a-34d13206b1b6 (Cited March 16, 2023).

[56] Zaror C Vergara-Gonzalez C Ibalaca N Olmos JP Perez S. Current state of teledentistry in Chile. J Int Soc Telemed EHealth. (2019) 7:e12(1–6). 10.29086/JISfTeH.7.e12

[57] Espinoza-Santander I Maturana-Ramírez A Hevia-Kulf MJ Sabando-Franulic V Letelier-Ruiz MJ Espinoza-Santander I “Célula de patología oral—hospital digital”: una estrategia de teleodontología para reducir desigualdades en el acceso a la atención odontológica en la especialidad de patología oral y maxilofacial en Chile/oral pathology cell—digital hospital”: a teledentistry strategy to reduce inequalities in access to dental care in the specialty of oral and maxillofacial pathology in Chile. Int J Interdiscip Dent. (2022) 15(2):114–5. 10.4067/S2452-55882022000200114

[58] Meza-Palma L Rosales-Salas J Meza-Palma L Rosales-Salas J. Protocolo de teleodontología para asistencia al paciente en el manejo de urgencia dental. Cuarentena COVID-19 (SARS-CoV-2). categorización Remota de urgencia dental y asistencia (C.R.U.D.A.)/teledentistry protocol for patient assistance in emergency dental management. Quarantine COVID-19 (SARS-CoV-2). Remote categorization of dental emergency and assistance (C.R.U.D.A.). Int J Odontostomatol. (2020) 14(4):529–37. 10.4067/S0718-381X2020000400529

[59] Flores-Torres MH Huerta-Gutierrez R Potter MB. Mhealth: experiences and opportunities for cancer research in Mexico. Salud Publica Mex. (2022) 64(1):111–4. 10.21149/13125 35438903

[60] Segura-Gaspar PL Atoche-Socola KJ. Teleodontología en tiempos de la COVID-19/teledentistry during COVID-19. Rev Cient Odontol. (2021) 9(2):e062. Available at: https://revistas.cientifica.edu.pe/index.php/odontologica/article/view/916. 10.21142/2523-2754-0902-2021-062 PMC10919822 38465278

[61] Revelo G. La teleodontología como alternativa de atención durante la pandemia por COVID-19/teledentistry as an alternative care during the COVID-19 pandemic. Odontol Sanmarquina. (2021) 24(3):299–303. 10.15381/os.v24i3.19433

[62] International Labour Organization. Egypt—law 151/2020 on the protection of personal data. (2020). Available at: https://www.ilo.org/dyn/natlex/natlex4.detail?p_lang=en&p_isn=111246&p_count=7&p_classification=01 (Cited March 16, 2023).

[63] Ahram Online E. Egypt follows up on telemedicine initiative, part of digital transformation plan (2022). Available at: https://english.ahram.org.eg/NewsContent/1/2/473607/Egypt/Society/Egypt-follows-up-on-telemedicine-initiative,-part-.aspx (Cited March 16, 2023).

[64] Fadl I. Chief, general agency of accreditation and health oversight: telemedicine saves $4.28 billion annually in healthcare costs. Amwal Alghad (2022). Available at: https://amwalalghad.com/2022/11/30/رئيس-الاعتماد-والرقابة-الصحية-التطبي/ (Cited March 16, 2023).

[65] Hassan R Saber SE Elsewify T. Financial impact of COVID-19 pandemic on endodontic clinics in Egypt: a questionnaire-based report. Eur Endod J. (2022) 7(2):135–42. 10.14744/eej.2022.15238 35786575 PMC9285999

[66] GlobeMed Egypt. Taking care of healthcare: Egypt launches the first telehealth consultation service. Available at: https://www.globemedegypt.com/content/globemed-egypt-launches-first-telehealth-consultation-service-egypt-1 (Cited March 16, 2023).

[67] Libyan Dental Association, Libyan Ministry of Health. Libyan guidelines for minimizing risk of Covid-19 transmission in dental clinics. Tripoli, Libya: Libyan Dental Association (2020). Available at: https://www.lmb.ly/corona/book/10/1591570481285263 (1).pdf (Cited March 16, 2023).

[68] Elhadi M Msherghi A Elhadi A Ashini A Alsoufi A Bin Alshiteewi F Utilization of telehealth services in Libya in response to the COVID-19 pandemic: cross-sectional analysis. JMIR Med Inform. (2021) 9(2):e23335. 10.2196/23335 33606654 PMC7919841

[69] Universita Di Pavia. Saha project. Available at: https://saha.unipv.it/ (Cited March 16, 2023).

[70] United Nations Development Programme. UNDP, Japan, the startup Speetar, and the Ministry of Health launch the first telemedicine initiative in Libya. 2020 Available at: https://www.undp.org/libya/press-releases/undp-japan-startup-speetar-and-ministry-health-launch-first-telemedicine-initiative-libya (Cited March 16, 2023).

[71] ITEdgeNews. Nigeria slumbers on national e-Health policy strategy (2017). Available at: https://www.itedgenews.africa/nigeria-slumbers-on-national-e-health-policy-strategy/ (Cited March 16, 2023).

[72] Uche I. Telemedicine in Nigeria: data protection considerations. Mondaq (2022). Available at: https://www.mondaq.com/nigeria/data-protection/1159640/telemedicine-in-nigeria-data-protection-considerations (Cited March 16, 2023).

[73] Labisi OE. The legal framework of telemedicine in Nigeria. [Ilishan-Remo, Ogun State]: Babcock University (2021). Available at: https://www.researchgate.net/publication/353664869_THE_LEGAL_FRAMEWORK_OF_TELEMEDICINE_IN_NIGERIA (Cited March 16, 2023).

[74] Eduoh T. Telemedicine and MHealth in Nigeria: the COVID-19 challenges. Medium (2020). Available at: https://eduohtherrie.medium.com/telemedicine-and-mhealth-in-nigeria-the-covid-19-challenges-51bdf6e76eb1 (Cited March 16, 2023)

[75] Ezeonwumelu IJ Obijiaku IJ Ogbueche CM, Cohort R 2020, Nwaozuru U. Healthcare provider-to-patient perspectives on the uptake of teleconsultation services in the Nigerian healthcare system during the COVID-19 pandemic era. PLOS Glob Public Health. (2022) 2(2):e0000189. 10.1371/journal.pgph.0000189 36962178 PMC10021919

[76] Ngwu CC Fadare AS Ene CK Adamu VE. Awareness and use of teledentistry among dental health care professionals at alex ekwueme federal university teaching hospital (AEFUTH), Abakiliki, Nigeria. Orapuh J. (2021) 2(2):e816. https://www.orapuh.org/ojs/ojs-3.1.2-4/index.php/orapj/article/view/63/42.

[77] Statista. Nigeria: number of dentists by gender (2022). Available at: https://www.statista.com/statistics/1260792/number-of-dentists-in-nigeria-by-gender/ (Cited March 16, 2023).

[78] Percept Actuaries and Consultants. Case notes at the frontier: five case studies of South African telemedicine providers (2021). Available at: https://percept.co.za/wp-content/uploads/2021/03/Telemedicine-Providers-Case-Study-Report.pdf (Cited March 16, 2023).

[79] Gulube SM Wynchank S. Telemedicine in South Africa: success or failure? J Telemed Telecare. (2001) 7(Suppl 2):47–9. 10.1258/1357633011937100 11747657

[80] Fortuin JB Naidoo S. Opportunities for teledentistry in South Africa. S Afr Dent J. 2015;70(8):342–6. Available from: http://www.scielo.org.za/scielo.php?script=sci_arttext&pid=S0011-85162015000800004&lng=en.

[81] Alabdullah JH Daniel SJ. A systematic review on the validity of teledentistry. Telemed J E Health. (2018) 24(8):639–48. 10.1089/tmj.2017.0132 29303678

[82] Estai M Kanagasingam Y Tennant M Bunt S. A systematic review of the research evidence for the benefits of teledentistry. J Telemed Telecare. (2018) 24(3):147–56. 10.1177/1357633X16689433 28118778

[83] Ngubane M. Telemedicine in South Africa: benefits and challenges. The Public News Hub (2018). Available at: https://www.publicnewshub.com/telemedicine-in-south-africa-benefits-and-challenges/ (Cited March 16, 2023).

[84] World Health Organization. Considerations for the provision of essential oral health services in the context of COVID-19. Interim guidance (2020). Available at: https://apps.who.int/iris/bitstream/handle/10665/333625/WHO-2019-nCoV-Oral_health-2020.1-eng.pdf (Cited March 16, 2023).

[85] Sorenson C Japinga M Crook H Mcclellan M. Building a better health care system post-Covid-19: steps for reducing low-value and wasteful care. NEJM Catal (2020). Available at: https://wahealthalliance.org/wp-content/uploads/2013/11/Choos- (Cited March 16, 2023).

[86] Medical and Dental practitioners Council of Zimbabwe. Policies and guidelines: policy on international telemedicine (2014). Available at: https://www.mdpcz.co.zw/the-public/policies-and-guidelines/ (Cited March 16, 2023).

[87] Coker O. Zimbabwe: Econet’s dial-A-doc under MDPCZ’s magnifying class. TechCabal (2015). Available at: https://techcabal.com/2015/03/09/zimbabwe-econets-dial-a-doc-under-mdpczs-magnifying-glass/ (Cited March 16, 2023)

[88] Gambanga N. Health authority advises public against econet dial a doc. Techzim (2015). Available at: https://www.techzim.co.zw/2015/03/health-authority-advises-public-against-econet-dial-a-doc/ (Cited March 16, 2023).

[89] SolidarMed. Zimbabwe. Available at: https://www.solidarmed.ch/en/zimbabwe (Cited March 16, 2023).

[90] Marongwe P Wasunna B Gavera J Murenje V Gwenzi F Hove J Transitioning a digital health innovation from research to routine practice: two-way texting for male circumcision follow-up in Zimbabwe. PLOS Digit Heal. (2022) 1(6):e0000066. 10.1371/journal.pdig.0000066 PMC9931231 36812548

[91] Dodoo JE Al-Samarraie H Alsswey A. The development of telemedicine programs in Sub-Saharan Africa: progress and associated challenges. Health Technol (Berl). (2022) 12(1):33–46. 10.1007/s12553-021-00626-7 34849325 PMC8613515

[92] Manyati TK Mutsau M. Exploring the effectiveness of telehealth interventions for diagnosis, contact tracing and care of Corona virus disease of 2019 (COVID19) patients in Sub Saharan Africa: a rapid review. Health Technol (Berl). (2021) 11(2):341–8. 10.1007/s12553-020-00485-8 33585154 PMC7870280

[93] Mchechesi I Simemeza TM Chikuwadzo B Keller M. Zimbabwe telehealth pilot program identifies high risk patients in an underserved population using point of care echocardiograms and digital electrocardiograms. J Am Coll Cardiol. (2021) 77(18):3024. 10.1016/S0735-1097(21)04379-5

[94] Commutalk News. MDPCZ announces telehealth/Telemedicine as Covid-19 response (2020). Available at: http://commutalk.co.zw/mdpcz-announces-telehealth-telemedicine-as-covid-19-response/ (Cited March 16, 2023).

[95] Ministry of Health and Child Care. Health digitisation adopted by Cabinet (2020). Available at: http://www.mohcc.gov.zw/index.php?option=com_content&view=article&id=416:health-digitisation-adopted-by-cabinet (Cited March 16, 2023).

[96] Ministry of Health and Child Care Z. MoHCC digitizing the health space. Available at: http://www.mohcc.gov.zw/index.php?option=com_content&view=article&id=349:mohcc-digitizing-the-health-space&catid=84&Itemid=435 (Cited March 16, 2023).

[97] Chawurura T Manhibi R van Dijk J van Stam G. ehealth in Zimbabwe, a case of techno-social development. In: Nielsen P Kimaro HC. (eds) Information and communication technologies for development. Strengthening southern-driven cooperation as a catalyst for ICT4D. ICT4D 2019. IFIP Advances in Information and Communication Technology. (2019) 551:15–26. 10.1007/978-3-030-18400-1_2

[98] Furusa SS Coleman A. Factors influencing e-health implementation by medical doctors in public hospitals in Zimbabwe. S Afr J Inf Manag. (2018) 20(1): 1–9. 10.4102/sajim.v20i1.928

[99] Khumalo NB Mnjama N. National standardisation for eHealth information initiatives in hospitals in Bulawayo, Zimbabwe. J S Afr Soc Arch. (2018) 51(0):170–93. Available at: https://www.ajol.info/index.php/jsasa/article/view/186207

[100] Yuan ZY. The current research status and thinking on the history of stomatology in modern China. Zhonghua Kou Qiang Yi Xue Za Zhi (Chinese J Stomatol). (2022) 57(8):861–6. 10.3760/cma.j.cn112144-20220613-00322 35970782

[101] Council GO of the CCC& the GO of the SC. Issuing outline of the program for health china 2030 (2016).

[102] Commission NH and FP. Notice on issuing the 14th five-year plan of National Health Informatization Development (2017).

[103] Commission NH. Report on National Health Informatization: Regional Hygiene and Hospital Informatization (2019).

[104] Peking University. Starting ceremony of PKU stomatological Hospital Joint Remote Consultation Platform and First Joint Remote Consultation Successfully Conducted (2012).

[105] Commission M and N. Notice of the General Office of the Ministry of Industry and Information Technology and National Health Commission on Organizing 5G+ Healthcare Pilot Programs Applications (2020).

[106] University W. Our hospital has started internet treatment services that provides free online diagnoses during COVID (2020).

[107] Dingyu T. Remote dentist for army hygiene companies. Xinhua News Agency (2021). Available at: http://www.xinhuanet.com/mil/2021-06/20/c_1211208088.htm (Cited 2023 Mar 16)

[108] Doctor Daily. “5G+ healthcare”|Remote diagnosis and treatment allows oral patients in remote areas to see a doctor. Sina News (2021). Available at: https://k.sina.com.cn/article_1343692175_50171d8f02700z5ae.html#/ (Cited March 16, 2023).

[109] Tencent News. The first time in China that Fujian completed 5G remote robot dental implant implantation (2022). Available at: https://new.qq.com/rain/a/20220927A0APWI00.html (Cited March 16, 2023).

[110] The Government of Hong Kong SAR- Innovation and Technology Bureau. HKSmart city blueprint (2021). Available at: https://www.smartcity.gov.hk/index.html (Cited March 16, 2023).

[111] Hospital Authority- Hong Kong. TeleHealth (pilot) (2021). Available at: https://www2.ha.org.hk/hago/en/features/appointment-related/telehealth (Cited March 16, 2023).

[112] Sinclair Communications. Introducing an international teledentistry brand to Hong Kong thorugh integrated PR solutions (2023). Available at: https://www.sinclaircomms.com/our-work/launching-the-revolutionary-clear-aligner-brand-in-hong-kong/ (Cited March 16, 2023).

[113] Li G-H Yu-Hang Lam W Kong H Hsung T-C Pelekos G Ling W-K Automatic site-specific multiple level gum disease detection based on deep neural network. 2021 15th International Symposium on Medical Information and Communication Technology (ISMICT) (2021); Xiamen, China: IEEE.

[114] The University of Hong Kong. HKU maxillofacial surgeons develop AI-based web tool for prediction of patients’ oral cancer risk (2022). Available at: https://www.hku.hk/press/news_detail_24370.html (Cited March 16, 2023).

[115] World Bank. Population, total—Iran, Islamic Rep. | Data (2022). Available at: https://data.worldbank.org/indicator/SP.POP.TOTL?locations=IR (Cited March 16, 2023).

[116] Taylor P. Iran mobile cellular subscriptions 2000–2020. Statista (2023). Available at: https://www.statista.com/statistics/498369/number-of-mobile-cellular-subscriptions-in-iran/ (Cited March 16, 2023).

[117] Shirmohammadi M Razeghi S Shamshiri AR Mohebbi SZ. Impact of smartphone application usage by mothers in improving oral health and its determinants in early childhood: a randomised controlled trial in a paediatric dental setting. Eur Arch Paediatr Dent. (2022) 23(4):629–39. 10.1007/s40368-022-00731-9 35841512 PMC9287817

[118] Lankarani KB Ghahramani S Zakeri M Joulaei H. Lessons learned from national health accounts in Iran: highlighted evidence for policymakers. Shiraz E Med J. (2015) 16(4):e27868. 10.17795/semj27868

[119] NZ Telehealth Forum & Resource Centre. About telehealth (2022). Available at: https://www.telehealth.org.nz/health-provider/what-is-telehealth/ (Cited March 16, 2023).

[120] Dental Council New Zealand. Roles and functions (2022). Available at: https://www.dcnz.org.nz/about-the-dental-council/what-we-do/roles-and-functions/ (Cited March 16, 2023).

[121] Dental Council New Zealand. Guidelines for using telehealth in dentistry during the COVID-19 alert level response (2020). Available at: https://www.dcnz.org.nz/assets/Uploads/COVID/Telehealth-during-COVID-19-response.pdf (Cited March 16, 2023).

[122] Polonowita A Guan G Thomson WM Martin-Hendrie R. Using telehealth for oral medicine patient management during the COVID-19 lockdown. Oral Surg. (2022) 15(3):291–304. 10.1111/ors.12721

[123] Lv N Sun M Polonowita A Mei L Guan G. Management of oral medicine emergencies during COVID-19: a study to develop practise guidelines. J Dent Sci. (2021) 16(1):493–500. 10.1016/j.jds.2020.07.016 32837687 PMC7413161

[124] Official Gazette Qatar. Law No. (22) of 2021 regulating healthcare services within Qatar. p. 42 (2021). Available at: https://almeezan.qa/LawView.aspx?opt&LawID=8761&language=ar

[125] Hukoomi Qatar e-Government. MoPH launches new remote healthcare services (2020). Available at: https://hukoomi.gov.qa/en/news/moph-launches-new-remote-healthcare-services (Cited March 16, 2023).

[126] Ali SA Al-Qahtani AMA Al Banai SR Albaker FJ Almarri AE Al-Haithami K Role of newly introduced teledentistry service in the management of dental emergencies during COVID-19 pandemic in Qatar: a cross-sectional analysis. Telemed J e-Health. (2022) 28(11):1623–32. 10.1089/tmj.2021.0584 35333637 PMC9700353

[127] Ministry of Health Saudi Arabia. Overview of E-Health (2021). Available at: https://www.moh.gov.sa/en/Ministry/nehs/Overview-of-eHealth/Pages/Overview-of-eHealth.aspx (Cited March 16, 2023).

[128] Ministry of Health Saudi Arabia. Telemedicine (2022). Available at: https://www.moh.gov.sa/en/Ministry/Information-and-services/Pages/Telemedicine.aspx (Cited March 16, 2023).

[129] Vision 2030 Saudi Arabia. Health sector transformation program. Available at: https://www.vision2030.gov.sa/v2030/vrps/hstp/ (Cited March 17, 2023).

[130] Ministry of Health Saudi Arabia. Medical consultation (Telehealth) (2022). Available at: https://www.my.gov.sa/wps/portal/snp/servicesDirectory/servicedetails/12423 (Cited March 16, 2023).

[131] Philips. 10 examples of telehealth apps that give a glimpse into the future of care. Available at: https://www.philips.sa/a-w/about/news/archive/standard/news/blogs/20210630-examples-of-telehealth-in-action-that-gives-a-glimpse-into-the-future-of-care.html (Cited March 16, 2023).

[132] Apple Saudi Arabia. The future of healthcare is in your hands. Available at: https://www.apple.com/sa-ar/healthcare/ (Cited March 16, 2023).

[133] Almazrooa SA Mansour GA Alhamed SA Ali SA Akeel SK Alhindi NA The application of teledentistry for Saudi patients’ care: a national survey study. J Dent Sci. (2021) 16(1):280–6. 10.1016/j.jds.2020.04.014 33384810 PMC7770252

[134] Al-Shammery D Alqhtani N Alotaibi AN AlSharidah M AlShehri K AlShamrani A. Contributions and concerns about the use of teledentistry in clinical orthodontics. Oral Health Prev Dent. (2021) 19(1):465–9. 10.3290/j.ohpd.b2081389 34585871 PMC11640954

[135] Kumar G Rehman F Al-Muzian L Farsi D Hiremath S. Global scenario of teledentistry during COVID-19 pandemic: an insight. Int J Clin Pediatr Dent. (2021) 14(3):426–9. 10.5005/jp-journals-10005-1952 34720519 PMC8543979

[136] Chaudhary FA Ahmad B Javed MQ Mustafa S Fazal A Javaid MM Teledentistry awareness, its usefulness, and challenges among dental professionals in Pakistan and Saudi Arabia. Digit Heal. (2022) 8:20552076221089776. 10.1177/20552076221089776 PMC8958680 35355810

[137] Al-Khalifa KS AlSheikh R. Teledentistry awareness among dental professionals in Saudi Arabia. PLoS One. (2020) 15(10):e0240825. 10.1371/journal.pone.0240825 PMC7561132 33057381

[138] Aboalshamat K Alkiyadi S Alsaleh S Reda R Alkhaldi S Badeeb A Attitudes toward social media among practicing dentists and dental students in clinical years in Saudi Arabia. Open Dent J. (2019) 13(1):143–9. 10.2174/1874210601913010143

[139] AlShaya MS Assery MK Pani SC. Reliability of mobile phone teledentistry in dental diagnosis and treatment planning in mixed dentition. J Telemed Telecare. (2020) 26(1–2):45–52. 10.1177/1357633X18793767 30134778

[140] AlKlayb SA Assery MK AlQahtani A AlAnazi M Pani SC. Comparison of the effectiveness of a mobile phone-based education program in educating mothers as oral health providers in two regions of Saudi Arabia. J Int Soc Prev Community Dent. (2017) 7(3):110–5. 10.4103/jispcd.JISPCD_95_17 28584780 PMC5452563

[141] Aboalshamat K. Awareness of, beliefs about, practices of, and barriers to teledentistry among dental students and the implications for Saudi Arabia vision 2030 and coronavirus pandemic. J Int Soc Prev Community Dent. (2020) 10(4):431–7. 10.4103/jispcd.JISPCD_183_20 33042884 PMC7523925

[142] Mahdavi A Atlasi R Naemi R. Teledentistry during COVID-19 pandemic: scientometric and content analysis approach. BMC Health Serv Res. (2022) 22(1):1111. 10.1186/s12913-022-08488-z 36050678 PMC9436727

[143] Cheuk R Adeniyi A Farmer J Singhal S Jessani A. Teledentistry use during the COVID-19 pandemic: perceptions and practices of Ontario dentists. BMC Oral Health. (2023) 23(1):72. 10.1186/s12903-023-02772-y 36739377 PMC9899062

[144] Lin GSS Koh SH Ter KZ Lim CW Sultana S Tan WW. Awareness, knowledge, attitude, and practice of teledentistry among dental practitioners during COVID-19: a systematic review and meta-analysis. Medicina (Kaunas). (2022) 58(1):130. 10.3390/medicina58010130 35056438 PMC8781277

